# Assessment of quality of care in a pediatric emergency unit of a tertiary hospital, Ethiopia

**DOI:** 10.1186/s12913-026-14100-5

**Published:** 2026-01-30

**Authors:** Muluwork Denberu, Amanuel M. Haile, Tamrat Endebu, Girma Taye, Lulu Muhe

**Affiliations:** 1 Department of Pediatrics and Child Health, College of Health Sciences, Addis Ababa University, Addis Ababa, Ethiopia; 2 School of Public Health, College of Health Sciences, Addis Ababa University, Addis Ababa, Ethiopia

**Keywords:** Quality of care, Indicators, Pediatric emergency, Low resource setting, Ethiopia

## Abstract

**Introduction:**

Despite the critical importance of pediatric care in low-resource settings, characterized by limited infrastructure, scarce resources, and high patient volumes, the quality of care provided in these environments remains poorly understood. This study aimed to assess selected quality of care indicators in the pediatric emergency unit (PEU) of Tikur Anbessa Specialized Hospital (TASH).

**Methods:**

A cross-sectional study was conducted among children aged 0–14 years who visited the PEU of TASH between January 1 and March 30, 2024. Participants were selected using a consecutive sampling technique. Data were collected using a quality indicator performance checklist, clinical record reviews, and a structured questionnaire administered to parents or guardians. Descriptive and correlation analyses were performed using SPSS version 26. Quality indicators were evaluated based on the combined framework of the Institute of Medicine’s (IOM) six quality domains—safety, effectiveness, patient-centeredness, timeliness, equity, and efficiency—and Donabedian’s structure–process–outcome model.

**Results:**

A total of 289 pediatric patients were included, with a median age of 48 months (IQR: 16.5–86.5 months). Key performance measurements showed a weight documentation rate of 97.6%, an unscheduled re-attendance rate of 13.5% within one month, a median laboratory turnaround time of 202 min (95% CI: 167–247), and an 11.1% in-house completion rate for imaging tests (specifically computed tomography scans). The healthcare-associated infection rate was 2.8%, caregiver-provider communication ranged from 77.5% to 92.7% across various aspects of care, and parent/guardian satisfaction was 84.1%. A significant negative correlation was found between parent/guardian satisfaction and residential address (*r* = − 0.120, *p* < 0.05).

**Conclusion:**

Strengthening diagnostic turnaround times, improving discharge and follow-up systems, and ensuring equitable access for children from rural areas are essential to enhancing the overall quality of pediatric emergency care.

**Supplementary Information:**

The online version contains supplementary material available at 10.1186/s12913-026-14100-5.

## Introduction

Assessing the quality of care in pediatric emergency units (PEUs) is critical, particularly in low-resource settings where constraints such as limited infrastructure, high patient volumes, and workforce shortages pose significant challenges to delivering effective, timely, and safe care [1]. In alignment with Sustainable Development Goal (SDG) 3—“Ensure healthy lives and promote well-being for all at all ages”—enhancing the quality of pediatric emergency services is vital to reducing preventable childhood deaths [2]. Ethiopia’s under-five mortality trends are above the SDG 3.2 target of ≤ 25 per 1,000 live births [3]. A significant proportion of preventable child deaths occur in hospitals, and 45% of child deaths in hospitals in low-resource settings could be prevented with basic emergency and critical care [4].

Globally, a variety of metrics have been developed to assess emergency care performance [5], based not only on global frameworks but also on their feasibility, relevance, and clinical significance in a resource-limited context [6]. Indicators such as weight documentation, time to first medical contact (FMC), laboratory and imaging turnaround times, documentation of warning signs, healthcare-associated infections (HAIs), unscheduled re-attendance, and parent satisfaction were prioritized because they are both measurable and actionable within existing hospital systems [5, 7–9]. These quality indicators are often assessed against established standards and benchmarks. For instance, weight documentation is a commonly reported metric, with some studies showing rates as low as 65.76% in pediatric emergency departments (PEDs) [9], despite clinical guidelines recommending adherence levels of 90% or higher to ensure safe and effective care [10]. Unscheduled re-attendance refers to patients returning to the emergency department within a defined timeframe after their initial visit. Re-attendance rates in pediatric emergency settings generally range from 3.9% to 4.3% [11]. A review of laboratory turnaround times (TAT) suggests that achieving a 90% completion rate for common laboratory tests in under 60 min is a desirable target, particularly in emergency contexts where prompt results are essential for effective patient management [12]. Further, to organize and interpret these metrics, studies applied two complementary frameworks of performance measures: the Institute of Medicine (IOM) quality domains and Donabedian’s structure-process-outcome model. The IOM’s six domains—effectiveness, safety, patient-centeredness, timeliness, efficiency, and equity—provide a comprehensive lens for evaluating the overall quality of care [13]. Donabedian’s model [14] supports this by categorizing quality indicators into structural inputs (e.g., equipment, staff), care processes (e.g., triage, documentation), and outcomes (e.g., re-attendance, caregiver satisfaction), enabling a more operational analysis of performance [15].

In high-income countries, particularly through initiatives like the Emergency Medical Services for Children (EMSC) program in the U.S., comprehensive frameworks have been established to measure the quality of pediatric emergency care [16]. These frameworks incorporate key quality metrics such as timely triage, pain assessment, imaging appropriateness, and return visits [16]. While these standards offer valuable guidance, they are often dependent on electronic health systems, advanced diagnostics, and specialized personnel—resources that are frequently unavailable, particularly in low-income countries (LICs). Furthermore, the epidemiology of pediatric emergencies in LICs differs markedly from that of high-resource settings, necessitating context-specific adaptations [17, 18]. Despite the urgent need for high-quality pediatric emergency care in LICs—where limited infrastructure, resource constraints, and high patient volumes are common—the quality of care in these settings remains inadequately studied and poorly understood.

Delivering high-quality pediatric emergency care requires not only adequate infrastructure and skilled staff but also timely care delivery, caregiver involvement, and routine quality monitoring [19]. In the context of Ethiopia’s ongoing health sector reform, there is a strong emphasis on enhancing the effectiveness, efficiency, and overall quality of healthcare services, particularly pediatric emergency services, to minimize child-related mortality [20]. National health strategies increasingly prioritize the routine measurement of key quality indicators as essential drivers of improvement across all levels of the health system [21].

Pediatric emergency units in tertiary referral hospitals, particularly in high-volume centers where the most complex and time-sensitive cases are managed, are critical entry points into Ethiopia’s health system; yet, evidence on the quality of care provided in these settings remains limited. Existing research in Ethiopia and sub-Saharan Africa has largely focused on adult emergency care or specific clinical outcomes, leaving a broader system-level assessment of pediatric emergency quality largely unexplored [22–25]. Therefore, this study aimed to assess selected quality-of-care indicators in the Pediatric Emergency Unit of TASH. Assessing quality in referral hospitals is especially important because these facilities function as national benchmarks, training sites, and centers of specialized expertise; thus, their performance has implications for the wider health system.

## Methods

### Study design and setting

An institution-based cross-sectional study was conducted at the Pediatrics Department of TASH, the largest tertiary referral hospital in Ethiopia (Addis Ababa), from January 1 to March 30, 2024. The department comprises a total of 183 pediatric beds, of which 45 are dedicated to the PEU. In 2024, the hospital received approximately 100,000 patient visits across all departments, with around 2600 pediatric patients seen in the emergency unit of TASH [26]. The unit mortality rate in the PEU was approximately 3.5%, and the overall pediatric department mortality rate was estimated at 1.5% during the same period [26]. The PEU was staffed by a multidisciplinary team that included 5 pediatric emergency physicians per shift, supported by general pediatric residents, pediatric nurses, emergency nurses, and rotating interns. Each shift typically includes 14 nurses and at least 2 senior residents, with access to on-call subspecialists as needed. The unit also serves as a teaching site for medical specialty training, as well as for postgraduate and undergraduate students, which further contributes to both the clinical workload and the unit’s educational responsibilities.

### Study population

The study population comprised all children aged 0–14 years who presented to the PEU of TASH during the study period. Children up to 14 years of age were included in accordance with departmental policy, whereby patients aged 15 years and older are managed in the adult emergency unit. The study population included neonates (1–29 days), infants (1–11 months), children (12–119 months), and adolescents (10–14 years). This categorization also aligns with the WHO definitions of childhood commonly used in pediatric emergency research [27].

### Inclusion and exclusion criteria

All children aged 0–14 years who presented to the PEU of TASH during the study period were eligible for inclusion. Children were excluded if they were dead on arrival, died shortly after arrival before data collection could be completed, were referred or transferred to another facility before clinical assessment, or if informed consent was declined by a parent or legal guardian.

### Sample size determination and sampling

The sample size for this study was calculated using the single population proportion formula, based on a 95% confidence level, a 5% margin of error, and a previously reported proportion of weight documentation (*p* = 0.658) [9]. Weight documentation was chosen as the reference indicator because it is one of the most frequently reported and easily measurable quality indicators in pediatric emergency departments. Moreover, it reflects both safety, through accurate medication dosing and monitoring of growth, and effectiveness, as it aligns with standard pediatric assessment protocols [28, 29]. Based on this, an initial sample size of 346 was estimated. After applying the Finite Population Correction due to the limited population size (*N* ≈ 1300) during the study period and adding a 10% non-response rate, the final adjusted sample size was 302. A consecutive sampling method was used, whereby every eligible case presenting during the study period was included until the sample size was reached.

For the assessment of parental satisfaction with the care provided, one parent or legal guardian per child was surveyed. The same sample size was applied to this component of the study.

### Data collection tools, quality indicators, and measurements

Data collection tools (checklists, record reviews, and questionnaires) were adapted from relevant literature [8, 29–31] and context-specific clinical guidelines [32] (Supplementary file 1). Quality indicators were selected based on feasibility, clinical relevance, and alignment with WHO standards and the literature [8, 9, 33]. They were organized using Donabedian’s structure–process–outcome model [14] and the IOM quality domains [13]. Each indicator was linked to a specific data source to ensure reliability and minimize bias. Structural indicators were obtained through facility-level records (staffing rosters, equipment inventories, and service availability logs) and direct observation; process indicators from patient charts, including electronic medical records (EMR), which contained clinical documentation generated during the child’s emergency visit, including triage and first contact times, weight documentation, laboratory and imaging turnaround time (TAT) and communication. Clinical outcome indicators were obtained from patient outcome records (disposition and mortality logs) and caregiver exit interviews. Doctors and nurses in the PEU were consulted during observation to validate workflow and triage practices. Data from all tools were triangulated to enhance validity and minimize observer bias. A face-to-face structured interview was conducted with the parent or guardian at the time of discharge using a questionnaire that included a 19-item measure of parent/guardian satisfaction. It was adapted from a validated survey originally developed for measuring parent satisfaction in a pediatric intensive care unit (reliability coefficient = 0.83) [34]. Although not originally designed for pediatric emergency settings, it was chosen because of its comprehensiveness and feasibility in our context. To generate an overall measure of the parent/guardian experience, a composite satisfaction score was calculated by averaging the 19 items rather than summing them [34]. This approach preserves the original 1–5 Likert response scale (1 = strongly disagree to 5 = strongly agree), facilitates more intuitive interpretation, and allows comparability across respondents when occasional item-level non-responses occur. The items covered six key domains of care: communication, care quality, family involvement, timeliness of care, emotional support, and the child-friendly environment. Parent/guardian respondents were considered proxy reporters of the child’s experience. The satisfaction assessment captures key dimensions of care, including communication, quality of care, family involvement, timeliness, emotional support, and the child-friendly environment. To ensure data quality, consistency, and minimize bias, two trained internists collected data using pre-tested tools. In addition, a supervising resident was assigned to oversee the entire data collection process. Measurements of the selected quality indicators are presented in Table 1.

**Table 1:** Definitions and measurements of quality indicators

Quality indicators	Data source	Definition and measurement
First medical contact (FMC) time	Patient charts and EMR	The time at which a patient is first assessed by a qualified healthcare professional in the emergency department [35]
Healthcare-associated Infections (HAIs)	Patient charts and EMR	Infections not present or incubating at the time of admission that develop during the hospital stay or within one incubation period after discharge [36].
In-hospital laboratory/imaging tests completion	Patient charts and EMR	Percentage of laboratory/imaging tests requested and performed in the hospital [35].
Laboratory Turnaround Time (TAT)	Patient charts and EMR	The time interval between the receipt of a specimen in the laboratory and the reporting of the test result to the clinician [12].
Return visits within 24 h leading to admission	caregiver interviews	The percentage of patients who return within 24 h and are subsequently admitted to the hospital [35].
Triage time	Patient charts	Is the time the patient was first assessed by the triage nurse upon arrival to the PED [37]
Turnaround time (TAT) in imaging	Patient charts and EMR	The time interval from when an imaging study (like an X-ray, CT, or MRI) is completed to when the final report is available to the ordering clinician [38].
Unscheduled re-attendance for the same diagnosis	caregiver interviews	The proportion of patients returning to the PED for the same diagnosis within a specified time of their initial visit [11].
Weight documentation	Patient charts and EMR	The percentage of patients with documented weight during their emergency visit [35].

Abbreviations: CBC= Count blood cell, CT= Computed tomography scans, CXR= Chest X-rays, ED= Emergency Department, ELE= Electrolyte, LFT= Liver failure test, MRI= Magnetic resonance imaging, PED= Pediatric Emergency Department, RFT= Renal failure test, TAT= Turnaround times, U/S= Ultrasounds, UA= Urine analysis EMR= Electronic Medical Record

### Data processing and analysis

Data were initially entered into a spreadsheet, then reviewed and cleaned before being exported to SPSS version 26 for further cleaning, coding, and analysis. Descriptive statistics were the main analytic approach, supplemented with Kendall’s tau-b correlation to assess associations between satisfaction scores and selected variables. Descriptive statistics were used to summarize the data: continuous variables were presented using medians and interquartile ranges (IQRs), while categorical variables were expressed as frequencies and percentages. Kendall’s tau-b correlation analysis was employed to assess the correlation between the ordinal parent/guardian satisfaction scores and selected socio-demographic characteristics, as well as median laboratory TAT. A p-value of < 0.05 was considered indicative of statistical significance. The parent/guardian-reported satisfaction measures were analyzed alongside quality indicators to assess the patient-centeredness dimension. A combined framework of IOM quality domains and Donabedian’s structure–process–outcome model was used to organize the results, facilitating a structured interpretation of performance indicators across both system-level and patient-centered dimensions of care.

### Ethical consideration

Ethical clearance was obtained from the Institutional Review Board (IRB) of the College of Health Sciences, Addis Ababa University. The research was conducted in compliance with the Declaration of Helsinki. All the study participants were informed about the objective and importance of the study. Written informed consent was obtained from parents or legal guardians for the review of their child’s clinical records and for participation in the caregiver satisfaction survey. In addition, assent was obtained from children aged 7 years and older, in accordance with ethical guidelines. Oral informed consent was obtained from participating health care providers.

## Results

### Socio-demographic characteristics


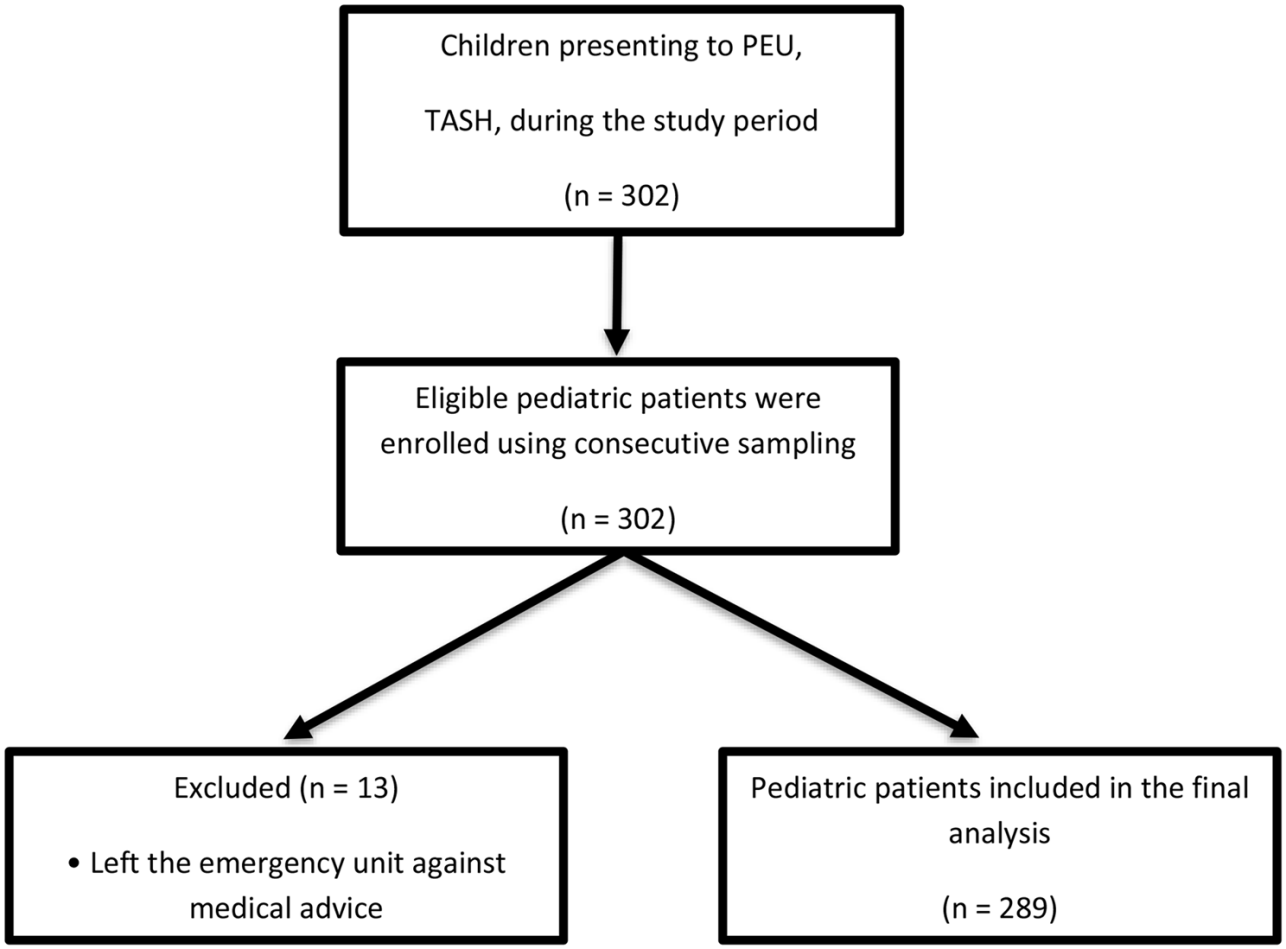


A total of 289 eligible pediatric patients were included in the study, each accompanied by a parent or guardian who completed the satisfaction survey. The median patient age was 48 months (IQR: 16.5–86.5). Among the participants attending the PEU, 11(3.8%) were neonates, 55(19.0%) were infants, 184(63.7%) were children, and 39(13.5%) were early adolescents aged 10–14 years. Of the pediatric patients, 169 (58.5%) were male, and 193 (66.8%) resided in urban areas. In terms of parental education, 149 (51.6%) of the fathers held a tertiary level, while 127 (43.9%) of the mothers were housewiv es. [Table 2].

**Table 2:** Socio-demographic characteristics among patients in a PEU of TASH, Ethiopia, 2022–2023 (*N* = 289)

Variables		Frequency	Percent		
Age in months					
Neonate (1–29 days)		11	3.8		
		Infant (1–11 months)		55	19.0
		Child (12–119 months)		184	63.7
		Adolescent (10–14 years)		39	13.5
Sex					
Male		169	58.5		
		Female		120	41.5
Address					
Urban		193	66.8		
		Rural		96	33.2
Education status of father					
No formal education		36	12.4		
		Primary school		54	18.7
		Secondary		50	17.3
		Tertiary (TVET/Diploma/higher)		149	51.6
Education status of mother					
No formal education		56	19.4		
		Primary school		64	22.2
		Secondary		79	27.3
		Tertiary (TVET/Diploma/higher)		90	31.1

### Measurements of quality-of-care indicators by age group

As shown in Table 3, weight was measured for nearly all pediatric patients, 100% of neonates, 98.2% of infants, 97.8% of children, and 94.9% of adolescents. Proper action before laboratory or imaging results was taken for over 90% across all ages, and warning signs were documented for 90.9% of neonates, 81.8% of infants, 79.9% of children, and 84.6% of adolescents. Unscheduled re-attendance within one month ranged from 9.1% among infants to 18.2% among neonates, while return visits within 24 h leading to admission were limited to children (4.3%) and adolescents (2.6%). Healthcare workers discussed the patient’s condition and diagnosis with most caregivers (≥ 90% across all ages), while discussions on treatment and next steps were slightly lower among children (around 75%). Written medication orders exceeded 94% in all groups, whereas daily progress note documentation was 81.8% among neonates and 53.8% among adolescents. Procedures were performed more often in children (15.2%) than in other groups, with full documentation among adolescents. HAIs occurred in 3.8% of children and 2.6% of adolescents, while none were reported among neonates or infants.

**Table 3:** Quality of care indicator measurements in the PEU of TASH by pediatric age group, expressed as column percentages, Ethiopia, 2022–2023 (*n* = 289)

Variable	Pediatric age					Total *N*(%)
	Neonate (1–29 days) (*n* = 11)	Infant (1–11 months) (*n* = 55)	Child (1–9 years) (*n* = 184)	Adolescent (10–14 years) (*n* = 39)	*P*-Value -	
Measured weight	11(100.0)	54(98.2)	180(97.8)	37(94.9)	0.569	282(97.6)
Measured height/length	9(81.8)	50(90.9)	165(89.7)	36(92.3)	0.712	260(90.0)
Known time of triage	11(100.0)	50(90.9)	172(93.5)	38(97.4)	0.579	271(93.8)
Known time to first medical contact (FMC)	0(0.0)	10(18.2)	22(12.0)	7(17.9)	0.291	39(13.5)
Failed to attend after registration	0(0.0)	0(0.0)	1(0.5)	0(0.0)	1.000	1(0.3)
Proper action taken before availability of imaging/laboratory results	10(90.9)	53(96.4)	174(94.6)	37(94.9)	0.705	274(94.8)
Documentation of warning signs given	10(90.9)	45(81.8)	147(79.9)	33(84.6)	0.815	235(81.3)
Rate of unscheduled re-attendance for same Dx within 1 month	2(18.2)	5(9.1)	26(14.1)	6(15.4)	0.674	39(13.5)
ED return visit within 24 h resulting in admission	0(0.0)	0(0.0)	8(4.3)	1(2.6)	0.502	9(3.1)
Health care worker discussed the patient’s condition	11(100.0)	53(96.4)	167(90.8)	37(94.9)	0.502	268(92.7)
Health care worker discussed diagnosis	11 (100.0)	52(94.5)	163(89.1)	36(92.3)	0.563	262(91.0)
Health care worker discussed possible treatment	10(90.9)	44(80.0)	139(75.5)	34(87.2)	0.592	227(78.5)
Health care worker discussed the next steps of management	11(100.0)	45(81.8)	136(73.9)	32(82.1)	0.137	224(77.5)
Patients documentation missed	0(0.0)	0(0.0)	7(3.8)	1(2.6)	0.575	8(2.8)
Written medication orders were given	11(100.0)	52(94.5)	176(95.7)	37(94.9)	0.857	276(95.5)
Progress note were written daily	9(81.8)	41(74.5)	124(67.4)	21(53.8)	0.320	195(67.5)
Procedure was done (e.g. LP, Ascitic tap, Pleural tap, Bone marrow aspiration)	2(18.2)	6(10.9)	28(15.2)	2(5.1)	0.392	38(13.1)
Procedure documented (*n* = 38)	0(0.0)	3(50.0)	16(57.1)	2(100.0)	0.357	21(55.3)
Any injuries/Iatrogenic problem occurs during service	0(0.0)	0(0.0)	3 (1.6)	0(0.0)	0.384	3(1.0)
Healthcare-associated infections (HAIs) were diagnosed	0(0.0)	0(0.0)	7(3.8)	1(2.6)	0.364	8(2.8)

Note: Fisher’s exact test was applied when expected cell counts were < 5. The final column presents the overall frequencies (n, %) representing the proportion of all children who received or experienced each quality-of-care component

### In-hospital laboratory testing and imaging performance

Among the requested laboratory tests, 97.9% of complete blood counts (CBC), 82.7% of renal function tests (RFT), 70.6% of liver function tests (LFT), 97.2% of electrolyte panels (ELE), 85.7% of blood films, and 100% of urinalysis (UA) and stool microscopy tests were completed within the hospital. Regarding imaging services, 88.7% of requested chest X-rays (CXR), 70.0% of ultrasounds (U/S), and only 11.1% of requested CT scans were performed in-hospital.

### Laboratory turnaround times (TATs)

The overall median turnaround time (TAT) for laboratory tests was 202 min (95% CI: 167, 246.99), with variations by test type: 45 min (IQR: 29, 103) for complete blood count (CBC), 203.5 min (IQR: 139, 300) for renal function tests (RFT), 209 min (IQR: 141.75, 335.25) for liver function tests (LFT), and 187.5 min (IQR: 120.75, 300) for electrolytes (ELE) **[**Table 4**].**

**Table 4:** Laboratory and imaging quality indicators in the PEU of TASH by setting of test completion (column %), Ethiopia, 2022–2023 (*n* = 289)

	Requested *n*(%)	Where it was done		Median TAT in Minutes (IQR)
		In Hospital *n*(%)	Out of Hospital *n*(%)	
The median time to FMC				*20* (95% CI: 12, 41)
*Laboratory tests*				
CBC	282 (97.6)	276 (97.9)	6 (2.1)	45 (29, 103)
RFT	104 (36.0)	86 (82.7)	18 (17.3)	203.5 (139, 300)
LFT	51 (17.6)	36 (70.6)	15 (29.4)	209 (141.75, 335.25)
ELE	142 (49.1)	138 (97.2)	4 (2.8)	187.5 (120.75, 300)
Blood film	14 (4.8)	12 (85.7)	2 (14.3)	300 (146, 375)
UA	82 (28.4)	82 (100.0)	0 (0.0)	77 (38.25, 160)
Stool microscopy	35 (12.2)	35 (100.0)	0 (0.0)	118 (43.75, 365)
*Overall Laboratory TAT*				*202 (69.25*,* 410)* (95% CI: 167, 246)
*Imaging*				
CXR	97 (33.6)	86 (88.7)	11 (11.3)	
U/S	90 (31.1)	63 (70.0)	27 (30.0)	
CT	18 (6.3)	2 (11.1)	16 (88.9)	
MRI	1 (0.3)	0 (0.0)	1 (100.0)	

CBC= Count blood cell, CT= Computed tomography scans, CXR= Chest X-rays, ELE= Electrolyte, LFT= Liver failure test, MRI= Magnetic resonance imaging, RFT= Renal failure test, TAT= Turnaround times, U/S= Ultrasounds, UA= Urine analysis

Note: ns represent the number of children for whom each test was requested. Values under “In-hospital” and “Out-of-hospital” represent the number (and percentage) of those tests performed in each setting

### Parent/guardian satisfaction and its correlation with demographics and lab TAT

The median parent/guardian satisfaction composite score was 4.16 (IQR: 3.84–4.47) on a five-point Likert scale [1–5], calculated as the average of 19 satisfaction items. Overall, 84.1% of respondents indicated agreement or strong agreement with the care provided to their children. Parents and guardians expressed the highest agreement with statements related to the adequacy of clinical care and communication, including trust in nurses treating their child (80.9% agree/strongly agree) and timely pain management (84.4%). Consistent and adequate information about the child’s condition and treatment plan also received high satisfaction ratings (> 85% agree/strongly agree across items). In contrast, aspects related to the physical environment scored comparatively lower. Only 51.5% of respondents agreed that the unit was quiet enough for the child to rest, and 72.0% felt that privacy and confidentiality were adequately respected during the visit (Supplementary file 3).

Parent/guardian reports were used as proxy measures for the child’s experience. Correlation analysis revealed a statistically significant negative association between parent/guardian satisfaction in the PED and their place of residence, with lower satisfaction reported among those from rural areas (Kendall’s tau-b = -0.120, *p* < 0.05). However, no significant correlations were found between parent satisfaction and median laboratory TAT (*r* = 0.052, *p* > 0.05), father’s education (*r* = 0.005, *p* > 0.05), or mother’s education (*r* = -0.001, *p* > 0.05) (Table 5).

**Table 5:** Correlation analysis between parent/guardian satisfaction and selected variables, Ethiopia, 2022–2023 (*n* = 289)

Variables		Parent satisfaction	Median Lab TAT	Address (rural)	Father’s education	Mother’s education
Parent satisfaction	r	1.000	0.052	**− 0.120** ^*^	0.005	− 0.001
	P-value	.	0.266	**0.032**	0.921	0.991
Median lab TAT	r	0.052	1.000	0.073	− 0.046	− 0.023
	P-value	0.266	.	0.137	0.315	0.604
Address (rural)	r	**− 0.120** ^*^	0.073	1.000	− 0.504^**^	− 0.495^**^
	P-value	**0.032**	0.137	.	0.000	0.000
Father’s education	r	0.005	− 0.046	− 0.504^**^	1.000	0.635^**^
	P-value	0.921	0.315	0.000	.	0.000
Mother’s education	r	− 0.001	− 0.023	− 0.495^**^	0.635^**^	1.000
	P-value	0.991	0.604	0.000	0.000	.

r = Kendall’s tau-b correlation coefficients; *. Correlation was significant at the 0.05 level (2-tailed);

**. Correlation was significant at the 0.01 level (2-tailed)

## Discussion

This study aimed to assess the quality of pediatric emergency care at TASH using frameworks, including the IOM quality domains and Donabedian’s structure–process–outcome model. The study revealed that a weight documentation, rate of 97.6%, an unscheduled re-attendance rate of 13.5% within a month, a rate of ED return visits within 24 h leading to admission (3.1%), a median time to first medical contact of 20 min (95% CI: 12–41), and a median laboratory turnaround time of 202 min (95% CI: 167–247). The majority of laboratory tests (70.6–97.6%) and common imaging tests like chest X-rays (88.7%) and ultrasounds (70.0%) were completed in-hospital, while 11.1% of CT scans were performed within the hospital setting. Similarly, communication with health care workers ranged from 77.5% to 92.7%, warning sign documentation in 81.3% of cases, and overall parent satisfaction was 84.1%. A statistically significant negative correlation was found between parent/guardian satisfaction in the PED and rural residents (*r* = − 0.120, *p* < 0.05). The results of important performance measures were summarized using both the IOM quality domains and Donabedian’s structure–process–outcome model (Supplementary file 4). In interpreting the findings, the IOM quality domains and Donabedian’s structure–process–outcome framework were primarily applied.

### Effectiveness

In this study, the weight documentation rate was 97.6%, classified under the effectiveness domain of the IOM framework and as a process indicator in Donabedian’s model. This finding was notably higher than weight documentation rates reported in previous studies conducted in other settings [28, 39]. The high documentation rate observed in this study may indicate effective processes for capturing weight information, which is essential for clinical decision-making. However, the accuracy of the recorded weight measurements was not assessed, which limits the interpretation of the documentation’s clinical reliability. Despite the high documentation rate, research indicates that dosing errors in pediatric care are common, occurring in 10% to 15% of cases, primarily due to inaccurate weight estimations or dosage miscalculations [40]. The literature underscores the urgent need for precise weight documentation in pediatric emergency settings to mitigate medication errors [28, 41]. Achieving 100% weight documentation with accurate estimations is vital for enhancing care effectiveness and ensuring patient safety, as it can significantly reduce the risk of errors related to weight-based medication dosing and other associated factors [29].

The rate of unscheduled re-attendance within one month for the same diagnosis—a key outcome indicator for measuring effectiveness in the PEU—was assessed based on Donabedian’s framework for healthcare quality [14] and other relevant literature [14, 42] —was found to be 13.5% in this study. This is notably higher than rates reported in similar studies conducted elsewhere [9, 11]. Ideally, re-attendance rates should range between 1 and 5% [43], and the elevated rate observed in this setting may reflect gaps in initial assessment, treatment, discharge planning, or overall quality of care [44]. Factors contributing to re-attendance in pediatric emergency departments may include progression of the original illness, missed or incorrect diagnoses during the initial visit, poor compliance with medication or discharge instructions, caregiver misunderstanding of the clinical advice provided, or new and unrelated health issues emerging after discharge [11, 45, 46]. A more detailed case review of re-attending patients would be valuable in identifying specific patterns or causes, which in turn could inform targeted quality improvement interventions. Such reviews could help to determine whether the re-attendance was avoidable and guide efforts to enhance clinical decision-making, caregiver communication, and discharge education [47] to reduce unnecessary revisits and improve overall patient outcomes.

### Timeliness

Time-related metrics, including time to triage and time to first medical contact (FMC), are essential components of emergency care. They enable the timely identification and treatment of critical conditions and help facilitate appropriate referrals or transfers when needed [8, 48]. In this study, the median time to first medical contact (FMC) was recorded at 20 min (95% CI: 12–41). While many guidelines recommend assessment within 15 min of arrival, this benchmark must be interpreted in the context of triage severity levels [49]. A study from a high-income setting validated recommended triage timelines in pediatric emergency departments, stating that medical care should be delivered immediately for level 1 patients, within 15 min for level 2, within 30 min for level 3, within 60 min for level 4, and within 120 min for level 5 patients [50]. Critically ill children, such as those in level 1 or 2 triage categories, require prompt evaluation and intervention, whereas children with lower-acuity conditions may wait longer without compromising care quality [49]. Therefore, the time to FMC is most meaningful when analyzed in relation to triage levels, as it provides a clearer picture of whether care was delivered according to clinical urgency. In our study, although the overall median FMC time was 20 min, the absence of stratification by triage category limits our ability to determine whether this time frame was appropriate for all patient acuity levels. Future assessments should incorporate triage data to evaluate whether timeliness benchmarks were met for each severity level, which would offer a more accurate reflection of emergency care responsiveness.

Laboratory turnaround time (TAT) is also a critical quality indicator in pediatric emergency units (PEUs), as delays in laboratory results can significantly impact clinical decision-making [12]. Timely access to diagnostic results enables early intervention, supports accurate diagnosis, and reduces the risk of complications or deterioration—particularly important in pediatric populations where conditions can progress rapidly [51]. This metric falls under the timeliness domain of the IOM framework and is classified as a process indicator within Donabedian’s model [7, 48]. In this study, the median laboratory TAT was 202 min (95% CI: 167–246), which is substantially longer than the 60-minute benchmark reported in previous studies from emergency care settings [9, 12, 52]. Several potential factors may contribute to the extended TAT, including delays in payment processing at the cash unit, overcrowding and patient flow challenges, particularly during peak hours, and staffing shortages [53, 54] and patient-level complexity, as pediatric cases often involve multiple concurrent symptoms, which can prolong diagnostic workflows [55]. Reducing laboratory TAT should be a strategic priority for emergency care improvement. This could involve optimizing workflow processes, increasing staffing during high-volume periods, digitizing cash and order systems, and implementing point-of-care testing where appropriate [54, 56].

### Efficiency

Lab and imaging test completion within a hospital can be categorized as a structural quality indicator in Donabedian’s model, reflecting the availability and accessibility of essential diagnostic services [14]. In this study, only 11.1% of requested CT scans were completed in-house, indicating limited internal capacity for advanced imaging. Although this finding was lower than a study reporting a 50% in-house CT scan rate [57], direct comparisons should be made cautiously due to differences in health systems, patient populations, resources, and case mix. The lower in-house completion rate observed in our setting may reflect unique contextual barriers, including limited access to CT equipment, operational constraints (e.g., hours of service or technician availability), or prioritization practices. We acknowledge a limitation: this study did not classify CT scan requests based on clinical urgency (i.e., emergent, urgent, or non-urgent), which limits the ability to fully assess whether delays or external referrals were appropriate. In spite of that, the current literature emphasizes the importance of implementing point-of-care diagnostic strategies, investing in imaging infrastructure and staff training, and strengthening internal referral and coordination systems [58].

### Safety

In this study, the rate of healthcare-associated infections (HAIs) was 2.8% during the study period. This finding was almost similar to other studies’ reports the rate of HAI ranges from 1.95% to 4.2% [59–61]. While this rate may appear low, even small percentages are clinically significant in emergency care, where rapid turnover, invasive procedures, and overcrowding can contribute to infection risks [62]. HAIs not only prolong hospital stays and increase healthcare costs but also contribute to morbidity, especially among neonates and immunocompromised children. The most common contributors to HAIs include lapses in infection prevention and control (IPC) practices, inadequate hand hygiene, overuse of invasive devices, and limited access to essential supplies such as disinfectants or personal protective equipment [62]. Strengthening IPC protocols, ensuring regular staff training, and implementing routine infection surveillance systems are essential steps toward reducing HAIs and improving patient safety in the pediatric emergency unit [63].

### Patient-centeredness

Caregiver-provider communication and parent satisfaction—representing process and outcome indicators of patient-centeredness—are essential for promoting high-quality, patient-centered care in PEUs [8, 33]. Although communication is directed toward the parent or guardian, its ultimate goal is to address the child’s needs by enabling caregivers to participate meaningfully in care decisions and manage the child’s care after discharge [64]. In this study, caregiver-provider communication ranged from 77.5% to 92.7% across various aspects of pediatric care, including discussions on the child’s condition, diagnosis, treatment options, and follow-up management. These results are consistent with previous studies that highlight communication as a central component of patient-centered care in pediatric emergency settings [65]. The item-level satisfaction results further highlight opportunities to strengthen patient-centered care in the PEU. While parents/guardians reported high satisfaction with communication and the quality of clinical care, lower ratings related to noise disturbance and privacy indicate that the physical and organizational environment may compromise the overall care experience. These areas align closely with WHO quality standards, emphasizing dignity, comfort, and confidentiality in pediatric emergency settings [66]. Likewise, the overall parent satisfaction rate was 84.1%, aligning with findings from similar settings [67] and exceeding those reported in other contexts [68]. While high satisfaction levels may reflect positive caregiver experiences, the use of face-to-face interviews to collect data may have introduced social desirability bias, potentially leading to overestimation of satisfaction rates. Importantly, targeted improvements—such as reducing crowding, optimizing patient flow, and enhancing environmental modifications to protect privacy—may contribute to better caregiver experience, improved trust in emergency services, and ultimately more efficient and safer care for children.

Despite the statistically significant negative correlation observed between parent/guardian satisfaction and rural residence, several underlying factors may explain this disparity. These findings are consistent with previous studies [25]. Barriers such as low health literacy [69] and socioeconomic disparities [70] can hinder effective communication and reduce satisfaction with care. Rural parents may also face longer wait times, limited follow-up options, and a disconnect with urban-centered care protocols, all of which can contribute to increased dissatisfaction [71]. Additionally, language differences present a challenge; in our study setting, rural parents often speak regional dialects, while healthcare providers primarily use the national language. Addressing these issues through culturally sensitive and linguistically appropriate communication strategies could help bridge the rural-urban satisfaction gap while promoting both safety and equity in pediatric emergency care [72, 73].

Overall, this study underscores both strengths and persistent gaps in the quality of pediatric emergency care at TASH. Process measures such as weight documentation and provider–caregiver communication performed well, whereas outcome indicators, including unscheduled re-attendance, prolonged laboratory turnaround times, and low in-house completion of advanced imaging, highlight areas for improvement. These findings should be viewed not merely as isolated performance measures but as reflections of broader systemic challenges in pediatric emergency care delivery. Addressing delays in diagnostic services, strengthening discharge and follow-up systems, and ensuring equitable access for rural families are critical steps toward enhancing the overall quality of care in the pediatric emergency unit. Importantly, the insights gained from this study extend beyond TASH and can inform national efforts to strengthen pediatric emergency care standards within Ethiopia’s broader health system. Future research should focus on developing and validating context-specific quality indicators and establishing routine monitoring mechanisms to guide policy and quality improvement initiatives across pediatric emergency settings.

### Strengths and limitations of the study

This study employed a comprehensive evaluation framework by integrating the IOM quality domains with Donabedian’s structure–process–outcome model, allowing for a multidimensional assessment of pediatric emergency care quality. Although WHO standards for improving pediatric care were not explicitly used as the primary framework, many of the indicators measured—such as timeliness, communication, and infection prevention—are closely aligned with WHO priorities for emergency care. However, several limitations should be considered. As a single-center study conducted in a tertiary referral and teaching hospital, the findings may not be fully generalizable to lower-level facilities or rural settings, although they are likely relevant to other high-volume referral hospitals facing similar resource and operational challenges in Ethiopia and comparable contexts. The reliance on descriptive analysis limits causal inference, and clinical outcomes were not directly measured. While face-to-face interviews minimized missing data, they may have introduced social desirability bias, and caregivers’ perceptions of communication and waiting times may be subject to recall bias—particularly following stressful or prolonged encounters. Additionally, equity considerations were not fully explored due to limited disaggregation by key sociodemographic variables. Finally, consecutive sampling and lack of adjustment for potential clustering (e.g., repeated visits by the same child) may have introduced bias. These factors should be taken into account when interpreting the study findings.

## Supplementary Information

Below is the link to the electronic supplementary material.

Supplementary Material 1

## Data Availability

Data are available from the corresponding author on reasonable request.

## Abbreviations

CBC: Complete Blood Count
CI: Confidence Interval
CT scan: Computed Tomography Scan
CXR: Chest X-Ray
ED: Emergency Departments
ELE: Electrolyte
FMC: First Medical Contact
HAI: Healthcare-Associated Infections
ICU: Intensive Care Unit
IOM: Institute Of Medicine
IQR: Interquartile Range
IRB: Institutional Review Board
LFT: Liver Function Test
MRI: Magnetic Resonance Imaging
PED: Pediatric Emergency Department
PEU: Pediatric Emergency Unit
RFT: Renal Function Test
SPSS: Statistical Package For Social Science
TASH: Tikur Anbessa Specialized Hospital
TAT: Turnaround Time
TVET: Technical and Vocational Education And Training
UA: Urine Analysis
U/S: Ultrasound
