# Supplementary material for: Assessment of quality of care in a pediatric emergency unit of a tertiary hospital, Ethiopia
Source: BMC Health Serv Res. 2026 Jan 30;26:297. doi: 10.1186/s12913-026-14100-5 (PMC12930605; doi:10.1186/s12913-026-14100-5)
Supplement: Supplementary file 1 — Supplementary Material 1 [file 12913_2026_14100_MOESM1_ESM.docx]

**SUPPLEMENTARY FILES**

**Supplementary file 1:** Checklist used to collect quality indicator performance data from clinical and record reviews, and structured questionnaires.

**I. Socio-demographic status**

1. Age in months _________________
2. Sex: Male
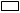
 Female
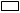

3. Address: Urban
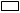
 Rural
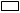

4. Education status of Father____________________________________
5. Education status of mother____________________________________
6. Occupation of the Father**_____________________________________**
7. Occupation of the mother**___________________________________________________**

**II. Quality indicator**

1. Weight Measured ___ Not measured ___
2. Height/Length Measured ___ Not measured ___
3. Time of triage _______________________ Unknown
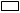

4. Time to first medical contact ____________________ Unknown
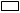


1. Failed to attend after registration a. yes
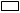
b. NO
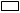
 Unknown
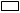

2. Laboratory turnaround time ( in min)

| **Lab investigation type** | **Not requested** | **Turnaround time** | **Unknown** |
| --- | --- | --- | --- |
| **CBC** |  |  |  |
| **RFT** |  |  |  |
| **LFT** |  |  |  |
| **ELE** |  |  |  |
| **Blood film** |  |  |  |
| **UA** |  |  |  |
| **Stool microscopy** |  |  |  |

1. Imaging turnaround time

| **Imaging type** | **Not requested** | **If Done, Where was it done?** | | **Imaging request time** | | **Imaging Film turnaround time** | | **Imaging reading result turnaround time** | |
| --- | --- | --- | --- | --- | --- | --- | --- | --- | --- |
|  |  | In Hospital | Out of hospital | Time of request | Unknown | turnaround time and date | Unknown | turnaround time and date | Unknown |
| **CXR** |  |  |  |  |  |  |  |  |  |
| **U/S** |  |  |  |  |  |  |  |  |  |
| **CT** |  |  |  |  |  |  |  |  |  |
| **MRI** |  |  |  |  |  |  |  |  |  |

1. Proper action taken before availability of imaging/Laboratory results yes
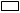
 No
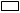
 Unknown
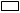

2. Duration of stay at PEU at the time of interview ________________ (in hours/days)
3. Documentation of warning signs given a. yes
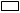
b. No
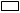
 Unknown
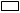

4. Rate of unscheduled re-attendance for same Dx within 1 month yes
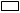
 No
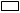
 Unknown
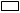

5. If “YES” for Q18 , How many times _____________________
6. ED return visit within 24 hours resulting in admission yes
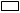
 No
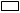
 Unknown
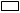

7. Patient-centeredness by effective partnerships between providers, patients
8. The health care worker discussed the patient condition a. yes
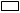
 b. No
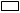
 Unknown
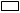

9. Health care worker discussed diagnosis a. yes
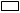
 b. No
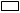
 Unknown
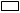

10. The health care worker discussed possible treatment a. yes
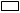
 b. No
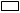
 Unknown
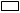

11. The health care worker discussed the next steps of management a. yes
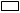
 b. No
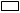


Unknown
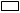


1. **Measurement of quality of care**
2. Patients documentation missed yes
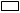
 No
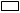
 Unknown
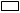

3. Is the medication written in the order sheet given to the patient yes
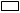
 No
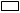
 Unknown
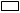

4. Is progress note written daily yes
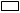
 No
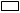
 Unknown
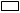

5. Was a procedure done yes
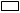
 No
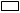
 Unknown
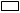

6. If “YES” to Q25 , What procedure was done LP
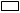
 Ascitic tap
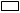
 Pleural tap
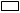
 Bone marrow aspiration
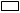
 Other
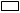

7. If “YES” to Q25, Is the procedure documented yes
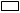
 No
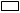
 Unknown
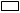

8. Are there any injuries/Iatrogenic problem occurs during service? yes
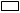
 No
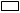
 Unknown
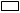

9. Is there any HAI yes
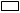
 No
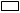
 Unknown
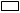

10. If”YES” to Q28 , How many days after admission is the HAI diagnosed ___________(in days)

1. **Parent/caregiver satisfaction measuring questionnaires**

Instruction: Please give one answer among the alternatives that most accurately reflects your view on each statement. The alternative answers are as follows

1. = Strongly disagree 2. = Disagree 3. = Neutral 4. = Agree 5. = Strongly agree

| Ser no | Questions | Responses options |
| --- | --- | --- |
| 1 | Did the care providers spend enough time with your child | 1. Strongly disagree  2. Disagree  3. Neutral  4. Agree  5. Strongly agree |
| 2 | Do you think your child has received excellent nursing care in the pediatric emergency? | 1. Strongly disagree  2. Disagree  3. Neutral  4. Agree  5. Strongly agree |
| 3 | Do you think your child has received excellent doctors care in the pediatric  Emergency? | 1. Strongly disagree  2. Disagree  3. Neutral  4. Agree  5. Strongly agree |
| 4 | Did the staff do everything they could do to help your child with his/her discomfort? | 1. Strongly disagree  2. Disagree  3. Neutral  4. Agree  5. Strongly agree |
| 5 | Did you have confidence and trust in nurses treating your child? | 1. Strongly disagree  2. Disagree  3. Neutral  4. Agree  5. Strongly agree |
| 7 | Were you allowed to be involved in your child's visit as much as you wanted? | 1. Strongly disagree  2. Disagree  3. Neutral  4. Agree  5. Strongly agree |
| 8 | Was there good communication between the different doctors and nurses? | 1. Strongly disagree  2. Disagree  3. Neutral  4. Agree  5. Strongly agree |
| 9 | Did you receive consistent information from all care providers during this visit? | 1. Strongly disagree  2. Disagree  3. Neutral  4. Agree  5. Strongly agree |
| 10 | Are you very satisfied with the care that your child has received in the pediatric emergency unit? | 1. Strongly disagree  2. Disagree  3. Neutral  4. Agree  5. Strongly agree |
| 11 | Would you recommend this pediatric unit to a friend or family member who needed to be treated | 1. Strongly disagree  2. Disagree  3. Neutral  4. Agree  5. Strongly agree |
| 12 | Do you think that your child has received adequate pain treatment within reasonable Period? | 1. Strongly disagree  2. Disagree  3. Neutral  4. Agree  5. Strongly agree |
| 13 | Does your child’s privacy and Confidentiality was respected during/this hospital stay? | 1. Strongly disagree  2. Disagree  3. Neutral  4. Agree  5. Strongly agree |
| 14 | Do you think your child’s room is quiet enough for him/her to rest? | 1. Strongly disagree  2. Disagree  3. Neutral  4. Agree  5. Strongly agree |
| 15 | Did you have the opportunity to participate in discussions concerning your child ‘examinations/treatments? | 1. Strongly disagree  2. Disagree  3. Neutral  4. Agree  5. Strongly agree |
| 16 | Have you had the opportunity to discuss the  goals of your child’s treatment with the  Child’s physician? | 1. Strongly disagree  2. Disagree  3. Neutral  4. Agree  5. Strongly agree |
| 17 | Do you think that the waiting time you have  Spent in the hospital without getting care reasonable? | 1. Strongly disagree  2. Disagree  3. Neutral  4. Agree  5. Strongly agree |
| 18 | Do you think that the child's needs have been taken care of, without waiting too long? | 1. Strongly disagree  2. Disagree  3. Neutral  4. Agree  5. Strongly agree |
| 19 | Do you think that you have received adequate information concerning your child's illness/course f illness? | 1. Strongly disagree  2. Disagree  3. Neutral  4. Agree  5. Strongly agree |
| 20 | Are you satisfied with how much the pediatric emergency doctors have told you about your child's expected outcome? | 1. Strongly disagree  2. Disagree  3. Neutral  4. Agree  5. Strongly agree |

Supplementary file 2

Sup. Table 1: Performance measurement framework organized by IOM quality domains (rows) and Donabedian’s structure–process–outcome categories (columns)

| **IOM quality domain** | **Donabedian framework** | | |
| --- | --- | --- | --- |
|  | **Structure** | **Process** | **Outcome** |
| **Effectiveness** |  | Weight documentation  Lab tests requested for blood film, UA, and stool microscopy.  Imaging requested for CXR, U/S, CT, and MRI. | Rate of unscheduled re-attendance for the same diagnosis within 1 month  ED return visit within 24 hours resulting in readmission |
| **Timeliness** |  | The median time to FMC  Laboratory median TAT  Imaging median TAT |  |
| **Efficiency** | Laboratory tests completed in hospital  Imaging tests completed in hospital |  | Failed to attend after registration |
| **Safety** |  | Proper actions before imaging/laboratory results  Warning signs documentation  Daily progress notes written  Written medication orders | Healthcare-associated infections (HAIs) |
| **Patient-centeredness** |  | Communication with health care workers (e.g., discussion of patient condition, diagnosis, possible treatments and next steps of management) | Parent/guardians overall satisfaction with the care received |

*CBC= Count blood cell, RFT= Renal failure test, LFT= Liver failure test, ELE= Electrolyte, UA= Urine analysis, CXR= Chest X-rays, U/S= Ultrasounds, CT= Computed tomography scans, MRI= Magnetic resonance imaging, TAT= Turnaround times*

**Supplementary file 3**

Sup. Table 2: Parent/guardian satisfaction with the quality of care in a PEU of TASH, Ethiopia, 2022-2023 (n=289)

| **SN** | **Variables** | **Strongly disagree** | **Disagree** | **Neutral** | **Agree** | **Strongly agree** |
| --- | --- | --- | --- | --- | --- | --- |
|  | **Information, communication, and education** |  |  |  |  |  |
| 1 | There good communication between the different doctors and nurses | 4(1.4) | 12(4.2) | 28(9.7) | 125(43.3) | 120(41.5) |
| 2 | They receive consistent information from all care providers during this visit | 4(1.4) | 6(2.1) | 29(10.0) | 116(40.1) | 134(46.4) |
| 3 | Thought that you have received adequate information concerning your child's illness/course f illness | 2(0.7) | 11(3.8) | 36(12.5) | 148(51.2) | 92(31.8) |
| 4 | Satisfied with their child's expected outcome | 2(0.7) | 10(3.5) | 39(13.5) | 145(50.2) | 93(32.2) |
|  | **Quality of care** |  |  |  |  |  |
| 5 | Thought their child has received excellent nursing care in the pediatric emergency | 3(1.0) | 21(7.3) | 39(13.5) | 159(55.0) | 67(23.2) |
| 6 | Thought their child has received excellent doctors care in the pediatric Emergency | 1(0.3) | 13(4.5) | 23(8.0) | 101(34.9) | 151(52.2) |
|  | **Respect preferences and involve the patient and family in care decisions** |  |  |  |  |  |
| 7 | Allowed to be involved in their child's visit as much as you wanted | 3(1.0) | 13(4.5) | 43(14.9) | 124(42.9) | 106(36.7) |
| 8 | Have the opportunity to participate in discussions concerning their child’s examinations/treatments | 2(0.7) | 11(3.8) | 50(17.3) | 133(46.0) | 93(32.2) |
| 9 | They had the opportunity to discuss the goals of their child’s treatment with the Child’s physician | 5(1.7) | 11(3.8) | 52(18.0) | 117(40.5) | 104(36.0) |
|  | **Timely and attentive care** |  |  |  |  |  |
| 10 | Care providers spend enough time with their child | 3 (1.0) | 14(4.8) | 41(14.2) | 139 (48.1) | 92 (31.8) |
| 11 | They thought that the waiting time they had spent in the hospital without getting care reasonable | 8(2.8) | 19(6.6) | 32(11.1) | 158(54.7) | 72(24.9) |
| 12 | They thought that their child's needs have been taken care of, without waiting too long | 5(1.7) | 19(6.6) | 33(11.4) | 156(54.0) | 76(26.3) |
|  | **Emotional support and pain management** |  |  |  |  |  |
| 13 | Have confidence & trust in nurses treating your child | 3(1.1) | 16(5.5) | 36(12.5) | 155(53.6) | 79(27.3) |
| 14 | Staff could help their child with his/her discomfort | 1(0.3) | 12(4.2) | 26(9.0) | 128(44.3) | 122(42.2) |
| 15 | Thought that their child has received adequate pain treatment within reasonable period | 0(0.0) | 10(3.5) | 35(12.1) | 165(57.1) | 79(27.3) |
|  | **Safe and child-focused environment** |  |  |  |  |  |
| 16 | Satisfied with the care that their child has received in the pediatric emergency unit | 2(0.7) | 11(3.8) | 40(13.8) | 138(47.8) | 98(33.9) |
| 17 | They would recommend this pediatric unit to a friend or family member who needed to be treated | 1(0.3) | 9(3.1) | 32(11.1) | 133(46.0) | 114(39.4) |
| 18 | Their child’s privacy and confidentiality was respected during/this hospital stay | 10(3.5) | 15(5.2) | 56(19.4) | 138(47.8) | 70(24.2) |
| 19 | Thought their child’s room is quiet enough for him/her to rest | 30(10.4) | 32(11.1) | 78(27.0) | 122(42.2) | 27(9.3) |
| *Overall parents/caregivers satisfaction* | | 0(0) | 14(4.8) | 32(11.1) | 174(60.2) | 69(23.9) |

**Supplementary file 4**

Summary of results organized by IOM quality domains (rows) and Donabedian’s structure–process–outcome categories (columns)

| **IOM Domain** | **Donabedian framework** | | |
| --- | --- | --- | --- |
|  | **Structure** | **Process** | **Outcome** |
| **Effectiveness** |  | - Weight documented: 97.6% - Height/Length documented: 90% - Lab tests requested: 97.6% for CBC; 36.0% for RFT; 17.6% for LFT; 49.1% for ELE; 4.8% for blood film; 28.4% for UA; and 12.2% for stool microscopy. - Imaging requested: 33.6% for CXR, 31.1% for U/S, 6.3% for CT, 0.3% for MRI. | - Rate of unscheduled re-attendance for the same diagnosis within 1 month: 13.5% - ED return visit within 24 hours resulting in admission: 3.1% |
| **Timeliness** |  | - The median time to FMC: 20 minutes (95% CI: 12, 41), i.e., percentage within target: 43.6% - Laboratory median TAT: 202 minutes (95% CI: 167, 246), i.e., percentage within target: 58.6% - Imaging median TAT: 173 minutes (95% CI: 132, 215) |  |
| **Efficiency** | - Laboratory Tests Completed in Hospital: CBC: 97.9%; RFT: 82.7%; LFT: 70.6%; ELE: 97.2%; Blood Film: 85.7% - UA: 100.0%;Stool Microscopy: 100.0% - Imaging Tests Completed in Hospital: CXR: 88.7%; US: 70.0%; CT: 11.1%; MRI: 0% |  | Failed to attend after registration: 0.3% |
| **Safety** |  | - Proper actions before imaging/laboratory results: 94.8% - Warning signs documented: 81.3% - Missed documentation: 2.8% - Daily progress notes written: 67.5% - Written medication orders: 95.5% | - Healthcare-associated infections (HAIs): 2.8% |
| **Patient-centeredness** |  | Communication with Health Care Workers:   - discussion of patient condition: 92.7%; - Discussion of diagnosis: 91.0%; - Discussion of possible treatments: 78.5%; - Discussion of next steps of management: 77.5% | Parent/guardians overall satisfaction with the care received: 84.1% |

*CBC= Count blood cell, RFT= Renal failure test, LFT= Liver failure test, ELE= Electrolyte, UA= Urine analysis, CXR= Chest X-rays, U/S= Ultrasounds, CT= Computed tomography scans, MRI= Magnetic resonance imaging, TAT= Turnaround times*
